# Improved control of exogenous attention in action video game players

**DOI:** 10.3389/fpsyg.2014.00069

**Published:** 2014-02-10

**Authors:** Matthew S. Cain, William Prinzmetal, Arthur P. Shimamura, Ayelet N. Landau

**Affiliations:** ^1^Department of Psychology, University of CaliforniaBerkeley, CA, USA; ^2^Visual Attention Lab, Brigham and Women’s Hospital, Harvard Medical SchoolCambridge, MA, USA; ^3^Ernst Strüngmann Institute for Neuroscience in Cooperation with Max Planck Society, Frankfurt am MainGermany; ^4^Department of Psychology, Hebrew University of JerusalemJerusalem, Israel

**Keywords:** individual differences, video game players, exogenous attention, attentional blink, cueing

## Abstract

Action video game players (VGPs) have demonstrated a number of attentional advantages over non-players. Here, we propose that many of those benefits might be underpinned by improved control over exogenous (i.e., stimulus-driven) attention. To test this we used an anti-cueing task, in which a sudden-onset cue indicated that the target would likely appear in a separate location on the opposite side of the fixation point. When the time between the cue onset and the target onset was short (40 ms), non-players (nVGPs) showed a typical exogenous attention effect. Their response times were faster to targets presented at the cued (but less probable) location compared with the opposite (more probable) location. VGPs, however, were less likely to have their attention drawn to the location of the cue. When the onset asynchrony was long (600 ms), VGPs and nVGPs were equally able to endogenously shift their attention to the likely (opposite) target location. In order to rule out processing-speed differences as an explanation for this result, we also tested VGPs and nVGPs on an attentional blink (AB) task. In a version of the AB task that minimized demands on task switching and iconic memory, VGPs and nVGPs did not differ in second target identification performance (i.e., VGPs had the same magnitude of AB as nVGPs), suggesting that the anti-cueing results were due to flexible control over exogenous attention rather than to more general speed-of-processing differences.

## INTRODUCTION

In the previous decade, action video game players (VGPs) have demonstrated a number of advantages over non-players (nVGPs) on visual and cognitive tasks. For example, VGPs have outperformed nVGPs on multiple object tracking (Green and Bavelier, 2006b), probabilistic inference (Green et al., 2010), forming detailed memory representations of objects (Sungur and Boduroglu, 2012), task switching (Cain et al., 2012), dual-task performance (Strobach et al., 2012), and multisensory integration (Donohue et al., 2010), among others (see Hubert-Wallander et al., 2011a for a review).

One aspect of video game experience that could underlie a variety of these benefits is control of attention, particularly control over exogenous attention. Action video games often have a great deal of visual distraction, so it would be plausible for VGPs to develop some level of control over the degree to which salient distractions in the visual environment capture their attention in order to promote better performance on their primary task. Consistent with this idea, VGPs have previously demonstrated reduced exogenous (i.e., stimulus-driven) attentional capture. In particular, VGPs were better able than nVGPs to avoid exogenous capture by task-irrelevant color-singletons in an additional singleton paradigm (Chisholm et al., 2010). VGPs were also better able than nVGPs to avoid exogenous capture by a suddenly appearing distractor in a color-singleton search (Chisholm and Kingstone, 2012). While this is strong evidence for improved distractor resistance in VGPs, other studies have demonstrated that VGPs use exogenous cuing to the same extent as nVGPs (Cain and Mitroff, 2011; Hubert-Wallander et al., 2011b). The key difference between these sets of studies is that in the experiments by Chisholm et al. (2010), Chisholm and Kingstone (2012) the potentially attention-capturing stimulus always indicated a to-be-ignored location (i.e., attending to it never aided task performance). Conversely, in the studies showing no differences in attentional capture between VGPs and nVGPs (Cain and Mitroff, 2011; Hubert-Wallander et al., 2011b), attending to exogenous cues would often have been beneficial to performance.

Previous work therefore suggests that a key difference between VGPs and nVGPs is the level of control over exogenous attentional capture: VGPs may exert control when exogenous attentional capture would hurt performance, but may not choose to exert control when capture would help or have no impact upon performance. Such flexibility could naturally arise from interaction with multiple action video games and multiple visual environments within such games and might affect performance in a wide variety of contexts outside of games. This notion is broadly similar to that put forward by Green et al. (2010) that VGPs are better than nVGPs at assessing and responding to the statistics of their visual environments and in line with evidence that VGPs may learn more quickly over the course of an experimental session (e.g., West et al., 2013).

**FIGURE 1:**
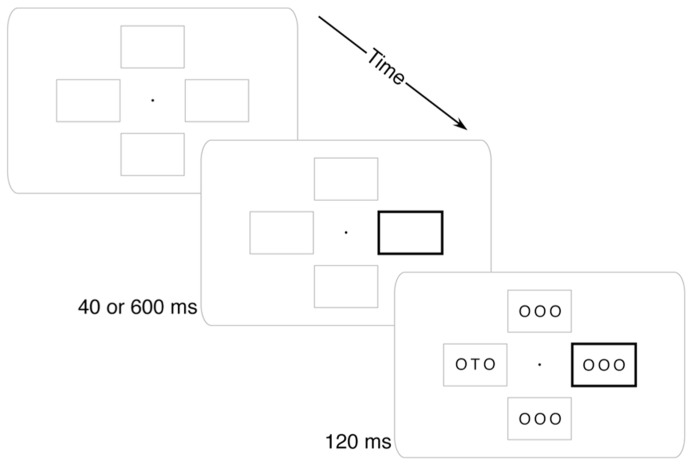
**Example trial.** Four placeholder boxes and a central fixation dot were always visible. At the beginning of each trial, one box would darken. After either 40 or 600 ms, the stimulus array would appear and participants would report whether a “T” or an “F” was present.

How flexible is VGPs’ avoidance of exogenous capture? Is it an all or nothing capacity, or can there be more graded control over exogenous attention? To address these questions we employ an *anti-cueing paradigm* (Experiment 1). In a typical spatial cuing task, there are specific locations where targets could appear and one of those locations is cued prior to target onset, generating exogenous capture. In target-cued conditions, the cue indicates the likely position of the target. In an anti-cueing paradigm, the appearance of the cue in one location actually indicates that a target will likely appear in a different location (Posner et al., 1982; Warner et al., 1990; Prinzmetal et al., 2009). For example, if the right location is cued (see **Figure 1**), there is a high probability that the target would appear on the left. Thus, the information given by the cue is task-relevant, but the spatial location of the cue is not the to-be-attended location. If VGPs can resist exogenous capture by this stimulus, but still use the information it provides in order to endogenously shift their attention, it would imply very precise control over attention.

In Experiment 2 we address the question of visual speed of processing using an attentional blink (AB) task. It has been argued that VGPs may process visual stimuli more quickly than nVGPs (e.g., Wilms et al., 2013). But is this faster apprehension related to overall processing-speed differences between VGPs and nVGPs? Might it even be associated with greater sensitivity to distractors (e.g., West et al., 2008)? If so, this could pose a problem for interpreting results showing reduced exogenous capture for VGPs, as attending to a stimulus and then very rapidly processing and disengaging from it may have the same behavioral effect as avoiding attentional capture at certain timescales.

To preview our results, we found superior control over exogenous attention in VGPs compared with nVGPs, but no differences between groups in endogenous attention or speed of processing.

## EXPERIMENT 1 – ANTI-CUE

In the anti-cue task, a cue is presented at one spatial location, but indicates that the target is likely to appear in a specific other location. This allows for the separation of the effects of exogenous attention and endogenous attention, a difference that should be more apparent in response time (RT) than in accuracy (Prinzmetal et al., 2009). If the sudden-onset of the cue exogenously captures attention, then when the interval between the cue and the target is short, participants should be faster to respond to those rare targets that appear at the location of the cue than those targets that appear in the more likely, anti-cued location. Conversely, when the interval between the cue and the target onset is longer, then participants will have sufficient time to endogenously move their attention to the likely target location, providing an advantage at the anti-cued location compared to the location of the cue. This design allows for separate assessments of the relative exogenous and endogenous attentional performance of VGPs and non-players.

### METHODS

#### Participants

Forty-two members of the University of California, Berkeley community participated in exchange for a cash payment or partial fulfillment of a course requirement. Other data from a subset of these participants that were collected in the same experimental session have been reported previously (Cain et al., 2012). Participants were recruited using a variety of methods including poster advertisements specifically seeking first-person shooting (FPS) game players and non-players and e-mail advertisements selectively sent to those with high and low levels of reported FPS expertise in a prescreening survey. Participants were not informed which survey in the prescreening packet lead to their recruitment until the end of the study.

Data from two participants were excluded, one for not completing the experiment and another for performing at chance-level accuracy throughout the experiment. The remaining 40 participants were classified into two groups based on their self-reported expertise and experience with action video games. The VGP group reported expertise with FPS video games of ≥5 on a 1–7 scale and regular play of FPS games (≥5 hr/wk) in the last 6 months. The VGP group consisted of 17 males and two females (mean age = 21.0 years). The non-player (nVGP) group reported expertise with FPS games of ≤2 on a 1–7 scale and recent experience with FPS games of <2 hr/wk in the last 6 months. Note that expertise or experience with other genres of video games (e.g., puzzle games) was not cause for exclusion from the nVGP group. The nVGP group consisted of eight males and 13 females (mean age = 22.5 years).

#### Stimuli

Four peripheral boxes and a central fixation dot were present on the screen throughout the experiment (see **Figure 1**). Each box extended approximately 2.0° × 1.25° and was 1 pixel thick. The innermost edge of each box was 1° from fixation. The fixation dot was a solid black circle 0.1° in diameter.

On each trial the cue was a thickening of the outline of one of the boxes to 0.1° wide. This thickened box remained visible until the stimulus array disappeared. The stimulus array included three characters per frame in a 36-point sans-serif font. The target letter was a “T” or an “F” and was always at the center of its array. All other placeholder letters in the display were “O”.

#### Procedure

The procedure is identical to that in Prinzmetal et al. (2009, Experiment 3). Participants were instructed to maintain fixation at all times during each trial. Fixation was monitored online using a video camera with a researcher labeling trials in which fixation was broken as they occurred. Eye movement trials were re-run at the end of the block in which they occurred.

On each trial a cue gave participants information about the likely position of the target. On 75% of trials the target appeared in the box opposite the cue (anti-cued location). On 12.5% of trials the target appeared in the same location as the cue (cued location). On the remaining 12.5% of trials the target appeared in one of the two off-axis boxes (other location); these catch trials were not included in any of the planned comparisons. Participants were informed that the target was “most likely” to appear in the anti-cued location, but could appear in any location. Participants were not given explicit probabilities.

The stimulus array appeared after the cue at one of two randomly intermixed stimulus onset asynchronies (SOAs). The Short SOA (40 ms) was intended to generate exogenous attention capture: participants should have had their attention drawn to the sudden-onset cue, but should not have had time to endogenously move their attention to the likely target (i.e., anti-cued) location. The Long SOA (600 ms) was intended to allow time for endogenous movement of attention from the cued location to the anti-cued location. The stimulus array remained on the screen for 120 ms (to minimize the utility of eye movements) at which time both the stimuli and cue disappeared. After the stimuli disappeared, participants responded whether a “T” or an “F” was present with a speeded keypress of the “1” and “2” keys on a numeric keypad using the index and middle fingers of their right hand.

Trials were presented in seven blocks, separated with self-paced breaks. The first block was 48 trials long, considered practice, and not analyzed. The six experimental blocks were each 96 trials long. Throughout the experiment, auditory feedback was given for incorrect responses and eye movements.

### RESULTS

**FIGURE 2:**
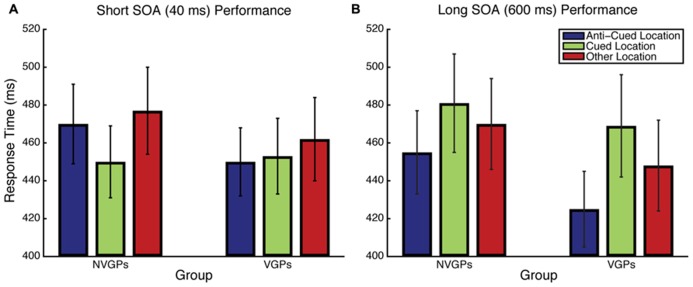
**Response time results for Experiment 1 for the Short SOA (A) and Long SOA (B) conditions.** Error bars represent the standard error of the mean.

Data from trials with RTs < 150 ms or > 1580 ms (three standard deviations above the mean RT for all correct trials) were excluded from analysis (0.9% of experimental trials). Analyses were conducted in parallel for both accuracy and RT (see **Table 1** for a full breakdown), with incorrect trials excluded from RT analysis. Data from the Other Location catch trials were not analyzed, but are reported in **Figure 2** and **Table 1** for comparison purposes.

**Table 1:** Breakdown of means and standard deviations (SDs) of accuracy and response time (RT) measures across all groups and conditions.

Short SOA (40 ms)								Long SOA (600 ms)					
Anti-cued				Cued		Other		Anti-cued		Cued		Other	
Measure	Group	Mean	SD	Mean	SD	Mean	SD	Mean	SD	Mean	SD	Mean	SD
Accuracy (%)	nVGP	95.6	3.6	95.8	4.9	95.5	6.4	95.7	4.3	96.4	3.7	95.8	4.0
	VGP	95.5	3.3	94.7	6.0	96.5	2.8	96.0	3.3	92.7	13.4	95.3	5.6
RT (ms)	nVGP	472	98	452	89	477	105	456	103	485	126	471	116
	VGP	451	80	453	88	462	96	426	89	469	117	451	112

*SOA, stimulus onset asynchrony; VGP, video game player; nVGP, non-video game player.*

#### Overall analysis

Results were primarily analyzed with linear mixed effects models (Baayen et al., 2008; Barr et al., 2013) using the lme4 package in R (Bates et al., 2013). These models are similar to repeated-measures ANOVAs, but use all experimental trials rather than averages and allow for better testing of proportional data (i.e., accuracy). For both accuracy and RT, models were constructed with Group (VGP or nVGP), Target Position (Cued or Anti-Cued), and SOA (40 or 600 ms) as fixed effects and Participant as a random effect. For accuracy, a logistic model that included a three-way Group × Target Position × SOA interaction fit the data significantly better than a model in which the Target × SOA interaction did not interact with Group [χ^2^(3) = 9.14, *p *= 0.0275]. Similarly for RT, a model that included a three-way Group × Target Position × SOA interaction fit the data significantly better than a model in which the Target × SOA interaction did not interact with Group [χ^2^(3) = 14.41, *p *= 0.0024]. To better understand how exogenous attentional capture varied between groups, we performed further analyses separately for each SOA. To preview, there was an interaction between Group and Target Position for RT, but not accuracy, in the Short SOA condition, and an interaction for accuracy, but not RT in the Long SOA condition.

#### Short SOA condition

Results for the Short SOA condition were analyzed using linear mixed effects models with Group and Target Position as fixed effects and Participant as a random effect. Accuracy was uniformly high and there was no difference between a logistic model that included a Group × Target Position interaction and one that did not [χ^2^(1) = 0.25, *p *= 0.6170]. RT results are summarized in **Figure 2A** and, unlike accuracy, showed evidence of a Group × Target Position interaction [χ^2^(1) = 4.73, *p *= 0.0296], implying that there are attentional cuing RT differences between groups. To understand the nature of this interaction, we performed *post hoc* paired-samples *t*-tests within each group. Consistent with previous findings, nVGPs were faster to respond when the target was at the cued location than at the anti-cued location [*t*(20) = 3.054, *p *= 0.006, Cohen’s *d *= 0.217]. However, VGPs were just as fast to respond to the target at the anti-cued location as at the cued location [*t*(18) = 0.417, *p *= 0.681, *d *= 0.030], suggesting reduced or eliminated exogenous attentional capture.

#### Long SOA condition

Results for the Long SOA condition were analyzed using the same linear mixed effects models as in the Short SOA condition. For accuracy, in contrast to the Short SOA condition, there was evidence of a Group × Target Position interaction [χ^2^(1) = 8.69, *p *= 0.0032]. To understand the nature of this interaction, we performed *post hoc* paired-samples *t*-tests on arcsine-square-root-transformed accuracy within each group. VGPs were more accurate when responding to targets at the anti-cued location and nVGPs were more accurate at responding to targets at the cued location, but neither of these individual comparisons was statistically significant (both *p *> 0.4). RT results are shown in **Figure 2B**. Unlike the Short SOA condition, there was no evidence of an interaction between Group and Target Position [χ^2^(1) = 0.08, *p *= 0.7813]. *Post hoc* paired-samples *t*-tests revealed that both groups showed significant cuing effects [VGPs: *t*(18) = 2.467, *p *= 0.024, *d *= 0.415; nVGPs: *t*(20) = 3.234, *p *= 0.004, *d *= 0.259].

### DISCUSSION

VGPs were better at resisting exogenous attentional capture by a suddenly appearing cue, but were just as able to use the information from the cue to endogenously direct their attention to a likely target location. Unlike the nVGP group, which demonstrated normal levels of attentional capture in the Short SOA condition, the VGP group performed equivalently quickly at all locations in the Short SOA condition. Importantly, in the Long SOA condition, the VGP group was able to use the cue to direct their attention to the probable target location, demonstrating the expected anti-cueing effect. Thus, the VGP group was not ignoring the task-relevant cue, but was able to suppress exogenous capture from its onset. Interestingly, a similar pattern of results has previously been shown with training on the anti-cue task (Warner et al., 1990), suggesting that general action video game experience may have a similar effect on underlying attentional mechanisms as specific task training.

There is an alternative explanation for the current results that bears consideration. It has been suggested that VGPs may enjoy a speed of processing advantage over nVGPs (Dye et al., 2009; Wilms et al., 2013). Perhaps the VGPs were experiencing just as much exogenous capture as the nVGPs, but were able to very rapidly process the cue, such that they were no longer captured by it when the target array appeared, even in the Short SOA condition. We address this speed of processing question in Experiment 2.

## EXPERIMENT 2 – ATTENTIONAL BLINK

Could the apparent resistance to exogenous capture seen in Experiment 1 be the result of faster processing of the cue stimulus? A few lines of evidence support this hypothesis. The most general claim is from a meta-analysis of VGP vs. nVGP studies that found that overall, VGPs perform faster than nVGPs with no loss in accuracy (Dye et al., 2009). This improvement could have come from increased speed of visual processing or from later stages such as decision processes, response execution, or some combination thereof. Other studies have demonstrated that VGPs are quicker to get information into visual working memory than nVGPs (Appelbaum et al., 2013) and are faster to accumulate visual evidence from noisy visual stimuli (Green et al., 2010). This suggests there may be a visual processing advantage for VGPs, but it’s not clear if this advantage would also apply to simpler situations like sudden-onsets. Most directly, one recent study specifically found faster visual processing for VGPs in a modified whole-report task (Wilms et al., 2013).

If faster visual processing in VGPs, lead to faster processing of the cue in Experiment 1, we might also expect faster processing of stimuli presented in quick succession in a rapid serial visual presentation task. In particular, VGPs would be expected to have a reduced *AB* (Raymond et al., 1992). The AB is a phenomenon where processing of one target item impairs processing of a second item encountered 200–500 ms later. This deficit is believed to be due to a processing bottleneck in which the second target cannot be processed simultaneously with the first target (see Martens and Wyble, 2010 for a review). If VGPs are faster at processing rapidly presented items, they may be able to more completely process the first target before the second appears, reducing the impact of this bottleneck and, thus, reducing the AB. Several previous studies suggest that VGPs have a reduced AB compared to nVGPs (e.g., Green and Bavelier, 2003; Oei and Patterson, 2013), though there is not complete agreement on this point (Boot et al., 2008; Murphy and Spencer, 2009). Importantly, not all AB tasks are the same (e.g., Kelly and Dux, 2011). Previous studies have used forms of the AB paradigm that involve other factors, such as task switching and fast apprehension of stimuli – two abilities previously shown to be superior in VGPs (e.g., Cain et al., 2012; Appelbaum et al., 2013). Here, we attempt to minimize the contributions of these other factors to better examine the question of speed of processing.

### METHODS

#### Participants

Fifty-two members of the University of California, Berkeley community participated in exchange for a cash payment or partial fulfillment of a course requirement, including 34 individuals who also participated in Experiment 1 as part of the same testing session. Other data from some participants have been reported previously (Cain et al., 2012). Data from three participants were excluded, one for making > 25% incorrect responses to first targets, and two for having incomplete data. Participants were divided into VGP and nVGP groups using the same criteria as for Experiment 1. The VGP group had 23 members (22 males and one female; mean age = 20.9 years) and the nVGP group had 26 members (11 males and 15 females; mean age = 22.2 years).

#### Stimuli and procedure

**FIGURE 3:**
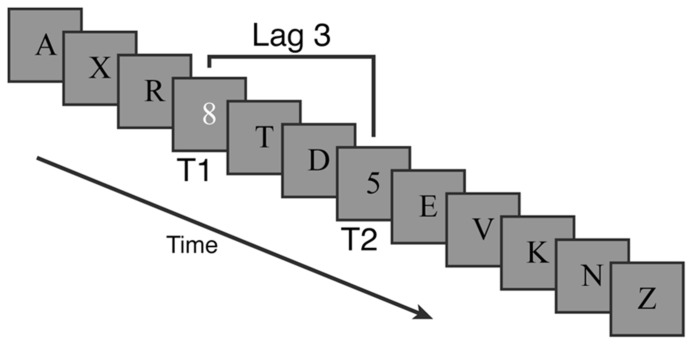
**Example trial for the attentional blink task in Experiment 2.** Targets were numbers among distractor letters. The first target was white and always present. The second target was black and present on 77% of trials.

Streams of letters (distractors) and numbers (targets) were presented at the center of the screen against a gray background (see **Figure 3**). Each trial’s stream contained 12 items presented for 80 ms each with a 20 ms inter-stimulus-interval (i.e., 100 ms stimulus onset asynchrony). Distractor items were black letters. Every trial contained a single white number target (T1) and 77% of trials contained an additional black number target (T2) that could only appear after T1. The remaining 23% of trials were catch trials that had no second target. Relative to T1, T2 could appear at lags of 1 (immediately after), 2, 3, 5, or 7 items.

On each of the 156 experimental trials participants observed the stream of characters and then separately reported the identity of the two target numbers using a standard computer keyboard. Participants used the space key to indicate that they did not see a particular number. Responses were unspeeded and instructions emphasized accuracy.

### RESULTS

**FIGURE 4:**
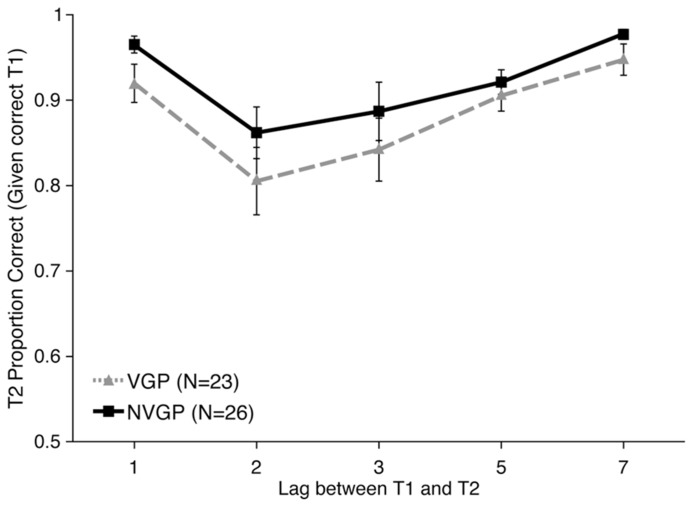
**Results from Experiment 2 showing second target accuracy for trials on which the first target was correctly identified as a function of inter-target Lag.** nVGPs non-significantly outperformed VGPs at all lags. Error bars represent standard error of the mean.

Accuracy data were analyzed for T2 on trials on which T1 was correct. First, T2 accuracy data were submitted to a linear mixed-model analysis with Lag (1, 2, 3, 5, or 7) and Group (VGP or nVGP) as fixed effects and Participant as a random effect. There was no evidence of an interaction between Group and Lag [χ^2^(4) = 4.6346, *p* = 0.3269]. Overall T2 accuracy was higher for nVGPs (92.4%) than VGPs (88.7%), but this Group difference was not statistically significant [χ^2^(5) = 6.4782, *p *= 0.2624]. As illustrated in **Figure 4**, this suggests that both groups experienced an AB, but that there were no differences between the groups. These models were followed up with *post hoc* *t*-tests comparing T2 performance between groups at each Lag and there were no significant differences at any point (all *p* > 0.05, uncorrected). There was no significant difference in T1 accuracy performance between groups [*t*(47) = 0.331, *p *= 0.743, *d *= 0.087].

#### Attentional blink magnitude

While there were no significant overall differences in performance between VGPs and nVGPs on this task, and nVGPs numerically outperformed VGPs, we wanted to specifically check AB performance. For each participant we calculated two AB scores: (1) Lag 7 (asymptote) performance minus Lag 2 (blink) performance and (2) the average of Lag 5 and Lag 7 minus the average of Lag 2 and Lag 3. For the Lag 7 minus Lag 2 measure, there was a significant overall AB effect of 13.39% [*t*(48) = 5.702, *p *< 0.001, *d *= 0.815], but no significant difference between groups [*t*(47) = 0.629, *p *= 0.532, *d *= 0.179]. The same pattern was seen for the average of Lags 5 and 7 minus average of Lags 2 and 3 measure: significant AB [*t*(48) = 4.764, *p *< 0.001, *d *= 0.6804], but no significant difference between groups [*t*(47) = 0.416, *p *= 0.679, *d *= 0.1180]. For both measures, VGPs had a numerically larger AB than nVGPs. While non-significant, this is noteworthy because it is opposite from the predicted direction.

### DISCUSSION

The current experiment demonstrated a robust AB effect, but no differences in performance between VGPs and nVGPs. If anything, nVGPs outperformed VGPs, the opposite of what was predicted based on previous work. This suggests two key points (1) that improved anti-cue performance for VGPs in Experiment 1 was due to improved resistance to attentional capture, rather than faster processing of the cue stimulus and (2) that improved performance was not due to general effects such as motivation or knowledge that the study was about video gaming (cf. Boot et al., 2011).

The lack of a difference between VGPs and nVGPs on this task stands in contrast to several previous reports. In particular, it contrasts with the initial finding by Green and Bavelier (2003; replicated in Oei and Patterson, 2013). While both our task and that of Green and Bavelier (2003) are considered to be AB tasks, and all AB tasks have significant shared variability (Dale et al., 2013), there are important differences between AB tasks that tap into task switching abilities and those that do not (Kelly and Dux, 2011; Dale et al., 2013).

In the present experiment, participants searched for numbers among letters. This is a categorical AB task that requires no task switching, since both targets are numbers to be detected among letters (T1 white, T2 black serially following T1). However, in Green and Bavelier’s (2003) experiment, participants had two different tasks to perform for the two embedded targets serially presented. First, they detected a white letter among black letters and then monitored for the presence or absence of an X. This probe-style AB task taps into task switching abilities as well as attentional selection abilities (Kelly and Dux, 2011). VGPs have been shown to switch between pairs of tasks on related stimuli more easily than nVGPs, including switching between letter and digit classification (Andrews and Murphy, 2006; Strobach et al., 2012), between global and local feature processing (Colzato et al., 2010), and between opposing stimulus-response rules (Cain et al., 2012). Thus, some of the video-game-related improvements in AB performance noted previously may have been due to superior task switching abilities in VGPs.

Additionally, in Green and Bavelier’s (2003) task, stimuli were presented very briefly (15 ms) while ours were presented relatively longer (80 ms). This presentation time difference likely contributed to the higher accuracy levels in our paradigm. In the 15 ms presentation version, the need to perceive the item quickly may have given the VGPs a further advantage, as VGPs have higher visual sensitivity than nVGPs and are better able to initially encode rapidly presented information into visual sensory memory (Appelbaum et al., 2013; but see Blacker and Curby, 2013; Wilms et al., 2013).

Thus, the superior performance seen in AB tasks previously may be due, in part, to improved task switching and visual sensitivity in VGPs relative to nVGPs and not to factors more commonly associated with the AB, such as the speed of processing T1. This idea of more general performance improvement is reinforced by an examination of the results of Green and Bavelier (2003), which shows a VGP advantage across Lags 1–5, and not just at the critical AB Lags and a training benefit at only later lags. While the current null result can provide only limited evidence, in combination with prior work, it suggests that the exact parameters of the AB task may be crucial for finding differences between VGPs and nVGPs.

## GENERAL DISCUSSION

Here we demonstrated that action VGPs have greater resistance to exogenous attentional capture than those who do not play action video games. In Experiment 1, when the time between the cue and the target was long, both VGPs and nVGPs showed the expected anti-cueing effect, responding faster at the anti-cued location than the cued location. Hence both groups displayed equivalent ability to utilize the information provided by the cue (i.e., predicting the anti-cue target location). However, when the SOA was short, nVGPs showed the expected exogenous cuing effect, but VGPs did not: nVGPs were faster at the location of the cue than at the most likely, anti-cued location, but VGPs were equally fast at all locations. Hence, while clearly extracting the information provided by the cue (as evident in longer SOAs) VGPs were able to avoid being captured to that same cue location. In Experiment 2, the finding that there was no difference in AB performance between VGPs and nVGPs suggests that the cuing effects were not due to speed of visual processing or motivational differences between groups.

These results are in line with recent findings that VGPs resist attentional capture by task-irrelevant distractors (Chisholm et al., 2010; Chisholm and Kingstone, 2012). However, it is seemingly at odds with a previous cuing finding: In a modified temporal-order judgment task with uninformative cues, VGPs were more likely to be captured by the cue than nVGPs (West et al., 2008; Experiment 1). The key difference between that paradigm and ours may be the informativeness of the cue. In the West et al. (2008) task, targets always appeared in both locations and the appearance of the cue carried no information about the relative target timings. Thus, from a participant’s point of view, attending to the cue had no noticeable effect on performance, so there was no particular reason to attempt to resist capture. In the current paradigm, the target only appeared in the cued location on 12.5% of trials, so being captured by the cued location might have noticeably negatively impacted performance, giving participants an incentive to try and resist capture. Also, we explicitly instructed participants that the target would most likely not appear in the cued location, and it may be that the VGP group was better able to use this instructional information than the nVGP group.

Our results fill in an important gap in the existing literature on attentional capture in VGPs. Previous work has demonstrated that VGPs are captured by exogenous cues that aid in task execution (Hubert-Wallander et al., 2011b) or have a non-obvious negative impact (West et al., 2008) but are able to resist capture by exogenous distractors that obviously hindered performance (Chisholm et al., 2010; Chisholm and Kingstone, 2012). Here we presented task-relevant information at a to-be-ignored spatial location and demonstrated that VGPs were able to resist attentional capture to an irrelevant spatial location while still being able to use cue information from that location to help them on the task. Taken together these results suggest that VGPs may possess more flexible control over what does and does not capture their attention: When a stimulus facilitates performance, VGPs can get the full benefit of letting it capture their attention, but when it hinders performance VGPs can resist capture.

### RELATIONSHIP WITH OTHER VISUAL ATTENTION PHENOMENA

One effect that has been much discussed in the video game literature is the flanker compatibility effect (i.e., distractor items surrounding a central target item speed responding if they are compatible with the target but slow responding if they are incompatible). If VGPs have better control over exogenous attention capture, this suggests that they might be less affected by the presence of incompatible flanking items in a display. In fact, initial reports argued that VGPs were actually more affected by incompatible flanking items than were nVGPs (Green and Bavelier, 2003, 2006a). However, subsequent reports have found equivalent levels of flanker interference in VGPs and nVGPs (Irons et al., 2011; Cain et al., 2012). While there is still some disagreement on this issue, it is clear that VGPs do not experience less flanker interference than nVGPs, which suggests some limits on their ability to control their attention. One potentially important difference between the cuing and flanker paradigms is the proportion of validly cued trials; in cases where VGPs have resisted stimulus capture, it was beneficial to do so most of the time, but in flanker experiments there is usually an even ratio of compatible trials (where capture helps) and incompatible trials (where it hinders), perhaps not providing sufficient incentive to exert control over exogenous capture. This line of argument suggests that studies manipulating cue validity may be able to more fully link these literatures.

Another attentional paradigm where VGPs have demonstrated benefits over nVGPs is multiple object tracking. In particular, VGPs are able to track more objects moving among distractors than nVGPs (Trick et al., 2005; Green and Bavelier, 2006b; Sungur and Boduroglu, 2012). This improved tracking performance is consistent with improved resistance to attentional capture: If VGPs are better able to resist capture by distracting items as those items pass near targets, this could lead to fewer instances where the target is lost. Unlike video game experience and training, specific spatial attention training does not lead to object tracking improvements (Appelbaum et al., 2011). This implicates a separate mechanism for superior performance by VGPs, such as exogenous attentional control.

### PROCEDURAL ISSUES

There has been increasing dialog about the best practices for studying the cognitive effects of video game experience (e.g., Boot et al., 2011; Kristjánsson, 2013), with two central issues: training vs. expert designs and participant recruitment. In the present experiments, we compared novice VGPs with expert VGPs. This has the advantage that our expert population has a great deal of experience (our VGPs reported playing ≥ 130 h of FPS games in the previous 6 months, between 2 and 10 times more exposure than in a typical training study), giving us the opportunity to observe skills that may only emerge after a great deal of practice. It should be noted, however, that such a quasi-experimental design has the drawback that we cannot be sure that the effects we observe are directly due to video game experience and not some other factor such as a selection bias (e.g., individuals with better control over attentional capture may play more FPS games, if such control makes gameplay more enjoyable).

One persistent source selection bias is gender, as action video games tend to engage males more than females (e.g., Lucas and Sherry, 2004). The present groups are not balanced by gender and thus, it is possible that gender differences in attentional abilities might underlie our effects (e.g., Feng et al., 2007), or the choices of our participants to become VGPs or nVGPs. A reanalysis of the current dataset including only male participants yielded the same general pattern of results, but the reduced statistical power limits the interpretability of this reanalysis. While we consider large differences in expertise with action video games between groups to be a more parsimonious explanation of the current results than gender differences, the current results are unable to definitively resolve this question.

Participants in these experiments were recruited both from prescreening survey responses and from fliers explicitly seeking VGPs and nVGPs. The explicit recruitment of some participants opens the possibility that groups were differently motivated, for example those identifying as VGPs may have come into the experiment expecting to perform well, while nVGPs may have had lowered expectations (e.g., Boot et al., 2011). While we cannot fully rule out this possibility, the lack of group differences in the AB task in Experiment 2, performed in the same testing session as Experiment 1, suggests that the effects were not driven solely by global motivational differences (see Cain et al., 2012; Schubert and Strobach, 2012 for similar arguments).

### CONCLUSION

There is no clear consensus on exactly what cognitive abilities are trained by action video game play or how such play actually leads to the generalized learning that has been observed. However, new ideas are beginning to emerge for how to characterize fundamental cognitive improvements due to video games (e.g., Baniqued et al., 2013). It seems clear that there are likely a number of factors that video games train, such as faster visual apprehension (e.g., Appelbaum et al., 2013), improved cognitive control (e.g., Cain et al., 2012; Strobach et al., 2012), and even the ability to quickly adapt within an experimental context (e.g., West et al., 2013). Here we argue that the ability to control and focus attention on task-relevant information is also a fundamental cognitive ability trained by video games. While the current study compared expert populations, and cannot speak directly about causality, one recent example more directly suggests a causal role. nVGPs were trained on custom FPS games that either required players to discriminate between hostile and friendly targets or contained exclusively hostile targets. Only those nVGPs in the target discrimination training condition showed attentional benefits from training (Brown et al., 2012).

The degree to which salient objects capture attention can vary from moment to moment (Leber, 2010). When acting in an uncertain visual environment, it would be advantageous to have flexible control over the level of exogenous attentional capture to a given location. Depending on the context, performance may be improved by allowing attention to be captured to a location by exogenous stimuli or by preventing capture. Action VGPs seem to be more adept than non-players at analyzing and adapting to the overall statistics of the visual task set at hand, likely due to extensive practice encountering, engaging with, and responding to the task demands of new environments in video games. In particular, the ability to extract information from a sudden-onset cue without allowing the cue to capture attention demonstrates a very high level of control over attention in VGPs.

## AUTHOR CONTRIBUTIONS

Matthew S. Cain and Ayelet N. Landau conceived of, executed, and analyzed both experiments. Matthew S. Cain wrote the manuscript. Arthur P. Shimamura and William Prinzmetal provided guidance and support. William Prinzmetal designed and programmed the task in Experiment 1.

## Conflict of Interest Statement

The authors declare that the research was conducted in the absence of any commercial or financial relationships that could be construed as a potential conflict of interest.
